# Mechanism of microRNA‐152 Regulating Decidual Natural Killer Cell Viability and Affecting Trophoblast Cell Invasiveness via the HLA‐G/KIR2DL4 Axis

**DOI:** 10.1002/kjm2.70019

**Published:** 2025-05-01

**Authors:** Yang Yang, Sai Liu, Xiao‐Ming Zhu, You‐Yi Chen, Jing Zhao, Yu‐Fei Yuan, Yuan Ma

**Affiliations:** ^1^ Reproductive Medicine Center Xi'an People's Hospital (Xi'an Fourth Hospital) Xi'an P. R. China; ^2^ Department of Obstetrics and Gynecology Hainan Branch of PLA General Hospital Sanya P. R. China; ^3^ Department of Obstetrics and Gynecology, Reproductive Medicine Center Tangdu Hospital, Air Force Military Medical University Xi'an P. R. China

**Keywords:** cytokines, decidual natural killer cells, HLA‐G/KIR2DL4, HTR‐8/SVneo cells, miR‐152

## Abstract

Trophoblast cells are specialized placental epithelial cells essential for pregnancy maintenance. miR‐152 is implicated in trophoblast cell regulation and pregnancy failure. This study explores the role of miR‐152 in decidual natural killer (dNK) cell viability and trophoblast cell invasion. HTR‐8/SVneo cells were transfected with miR‐152‐mimics/inhibitor or their respective controls, followed by co‐culture with dNK cells. RT‐qPCR assessed transfection efficiency, while cytokine secretion (IL‐8, IP‐10, VEGF), cell viability, apoptosis, and invasion were evaluated via ELISA, CCK‐8, flow cytometry, Western blot, and Transwell assays. The interaction between miR‐152 and HLA‐G was examined via dual‐luciferase reporter assay, and HLA‐G/sHLA‐G levels were measured. Co‐cultures of dNK cells and miR‐152/HLA‐G‐overexpressing HTR‐8/SVneo cells were established, and anti‐KIR2DL4/IgG1 was used to block HLA‐G/KIR2DL4 binding. Co‐immunoprecipitation confirmed protein interactions. miR‐152 overexpression suppressed dNK cell cytokine secretion, reduced HTR‐8/SVneo cell viability and invasion, and promoted apoptosis. miR‐152 inhibition had the opposite effect. miR‐152 directly targeted HLA‐G, and HLA‐G overexpression rescued dNK function and trophoblast invasion. Blocking the HLA‐G/KIR2DL4 binding counteracted the effects of miR‐152. miR‐152 inhibits dNK cell function and trophoblast invasion by targeting HLA‐G, reducing HLA‐G/KIR2DL4 interaction. These findings highlight a potential regulatory mechanism in pregnancy maintenance.

## 1 Introduction

Pre‐eclampsia (PE) is a gestational idiopathic disorder characterized by hypertension and proteinuria in pregnant women, making it a leading cause of maternal and perinatal mortality [1, 2]. In the United States, PE prevalence has steadily increased over the past two decades [3]. Various factors contribute to PE onset, including impaired maternal vascular remodeling, dysregulated spiral artery transformation, and oxygen imbalance [4]. Among these, trophoblast cell invasion and vascular remodeling are essential for proper placental development, and disruptions in these processes are considered contributors to PE [5, 6]. Despite these insights, the precise etiology and pathophysiology of PE remain to be fully elucidated.

Natural killer (NK) cells constitute the predominant immune cell type in the decidua during early pregnancy, accounting for approximately 70% of local lymphocytes. Unlike conventional NK cells, decidual NK (dNK) cells exhibit reduced cytotoxicity and secrete factors, such as vascular endothelial growth factor (VEGF), interferon‐inducible protein (IP)‐10 and interleukin (IL)‐8 to facilitate placental formation, trophoblast invasion, embryonic development, and vascular remodeling [7]. Given their fundamental role in these processes, dNK cell dysfunction is increasingly implicated in PE development.

A growing body of evidence suggests that dNK cells interact with human leukocyte antigen (HLA) ligands (HLA‐G, HLA‐C and HLA‐E) on extravillous trophoblasts (EVTs) to establish immune tolerance [7]. Among these, HLA‐G, a non‐classical HLA class‐I molecule exclusively expressed in EVTs, is crucial for protecting trophoblasts from NK cell‐mediated cytotoxicity [8]. This interaction is also essential for dNK cell differentiation and functional maturation, facilitating immune adaptation during pregnancy [9]. Notably, reduced HLA‐G expression at the maternal‐fetal interface has been associated with PE, with several cases exhibiting particularly diminished HLA‐G levels [10].

At the maternal–fetal interface, dNK cells express high levels of inhibitory killer immunoglobulin‐like receptors (KIRs), which recognize HLA‐G and suppress NK cell cytotoxicity [8]. Under normal physiological conditions, the KIR2DL4 receptor on dNK cells binds to HLA‐G, preventing an immune attack on the fetus. However, in PE, downregulation of HLA‐G disrupts this protective mechanism, leading to an immune‐mediated “missing‐self” response, where dNK cells become cytotoxic, contributing to placental dysfunction [11]. Additionally, KIR2DL4 binding to soluble HLA‐G (sHLA‐G) enhances proangiogenic and proinflammatory responses, essential for endometrial receptivity [12]. Guo et al. reported that reduced KIR2DL4 expression in dNK cells, coupled with lower HLA‐G levels in trophoblasts, hampers trophoblast invasion and angiogenesis, highlighting a potential mechanism underlying recurrent pregnancy loss [13]. Moreover, HLA‐G5 interaction with KIR2DL4 activates the ERK signaling pathway, promoting trophoblast invasiveness [14]. These findings suggest that the HLA‐G/KIR2DL4 axis is a crucial regulator of placental development and a promising target for PE intervention.

MicroRNAs (miRNAs), a class of small non‐coding RNAs, are key post‐transcriptional regulators that influence numerous biological functions, including immune responses and placental development. Among them, miR‐152 has been identified as significantly upregulated in the serum and placenta‐derived exosomes of PE patients, as well as in placental tissues of PE animal models, where it promotes trophoblast apoptosis [15]. Additionally, miR‐152 has been shown to directly target the 3′ untranslated region (3′UTR) of HLA‐G, reducing its expression and consequently preventing LILRB1‐mediated inhibition of NK cell cytotoxicity [16]. However, whether miR‐152 also impairs trophoblast invasion and dNK viability through the HLA‐G/KIR2DL4 remains unclear. In this study, we hypothesized that miR‐152 downregulates HLA‐G, thereby disrupting HLA‐G/KIR2DL4 interaction, impairing dNK cell, and ultimately inhibiting trophoblast invasion, potentially contributing to PE pathology.

## 2 Methods

### 2.1 Cell Culture

Human chorionic trophoblast cells (HTR‐8/Svneo, Cat. No. CRL‐3271) were obtained from the American Type Culture Collection (Manassas, VA, USA) and authenticated by STR. The cells were cultured in Roswell Park Memorial Institute‐1640 (RPMI‐1640) medium (Thermo Fisher Scientific, Waltham, MA, USA) supplemented with 10% fetal bovine serum (FBS) (Invitrogen, Carlsbad, CA, USA) at 37°C with 5% CO_2_.

### 2.2 Cell Transfection

HTR‐8/SVneo cells were grouped as below: the Control group (untreated cells), mimics NC group (HTR‐8/SVneo cells transfected with mimics NC for 48 h), miR‐152 mimics group (HTR‐8/SVneo cells transfected with miR‐152 mimics for 48 h), inhibitor NC group (HTR‐8/SVneo cells transfected with inhibitor NC for 48 h), miR‐152 inhibitor group (HTR‐8/SVneo cells transfected with miR‐152 inhibitor for 48 h), miR‐152 mimics + oe‐NC group (HTR‐8/SVneo cells co‐transfected with miR‐152 mimics and oe‐NC for 48 h), miR‐152 mimics + oe‐HLA‐G group (HTR‐8/SVneo cells co‐transfected with miR‐152 mimics and oe‐HLA‐G for 48 h).

miR‐152 mimics, miR‐152 inhibitor, and pcDNA3.1‐HLA‐G (oe‐HLA‐G), along with their respective controls mimics NC, inhibitor NC, and pcDNA3.1‐NC (oe‐NC) were procured from GenePharma (Shanghai, China). HTR‐8/SVneo cells in the logarithmic growth stage were plated in a 6‐well plate (1 × 10^6^ cells per well) and incubated overnight [13]. At 50%–60% confluence, cells were transfected with Lipofectamine 2000 (Invitrogen) following the manufacturer's protocol, using a final transfection concentration of 50 nM. Experimental analyses were performed 48 h post‐transfection.

### 2.3 Isolation and Purification of Primary dNK Cells

The decidual tissues were collected from healthy women undergoing pregnancy termination for non‐medical reasons (6–9 weeks of gestation) at Xi'an People's Hospital (Xi'an Fourth Hospital). As previously stated [13, 17], decidual cells were isolated using an enzyme dispersion method. Tissues were washed twice in phosphate‐buffered saline and minced into small fragments, and digested in RPMI 1640 medium (Thermo Fisher Scientific) containing 0.01 mg/mL DNase I (MP Biomedical, Santa Ana, CA, USA) and 1 mg/mL collagenase type IV (MP Biomedical) at 37°C for 40 min. The cell suspension was filtered through a nylon mesh, centrifuged, and the supernatant was discarded. Decidual monocytes were isolated using Ficoll density gradient centrifugation (MP Biomedical) and immediately processed for flow cytometry (FCM) and further analysis. dNK cells were positively selected using CD56 Microbeads (Miltenyi Biotec, Bergisch Gladbach, Germany) and stained with FITC‐labeled anti‐human CD56 antibody (562,794, BD Biosciences, San Jose, CA, USA), PE anti‐human CD3 antibody (980,008, BD Biosciences) and PE‐labeled anti‐human CD16 antibody (556,619, BD Biosciences). Cells were incubated at 4°C for 30 min, followed by fluorescence‐activated cell sorting, yielding a CD56^+^CD16^−^CD3^−^ cell population with more than 97% purity. These purified dNk cells were then used for subsequent experiments.

### 2.4 Methods of Cell Co‐Culture and Cell Re‐Isolation

As previously reported [13], a dNK cell (5 × 10^5^) and HTR‐8/SVneo cell co‐culture system was established by seeding 5 × 10^5^ cells of each type in 1 mL of RPMI 1640 medium supplemented with 10% IL‐15‐free FBS in a 24‐well plate. The co‐culture system was classified into the following groups: Control + dNK group, mimics NC/miR‐152 mimics + dNK groups, inhibitor NC/miR‐152 inhibitor + dNK groups, miR‐152 mimics + oe‐NC/oe‐HLA‐G + dNK groups, miR‐152 inhibitor + anti‐KIR2DL4/IgG1 + dNK groups. Anti‐KIR2DL4 (GTX80038, GeneTex, Irvine, CA, USA) was used to block the interaction between HLA‐G and KIR2DL4. A non‐specific homologous IgG1 (GTX35014, GeneTex) was used as a control at a final concentration of 10 μg/mL in RPMI 1640 medium.

Cell re‐isolation method: HTR‐8/SVneo cells and dNK cells were separated following 72 h of co‐culture. Suspended dNK cells were carefully aspirated using a pipette and transferred to a 1 mL EP tube. To enhance dNK cell recovery, the co‐culture plate was washed three times with fresh medium, and the washing dilution containing residual suspended cells was collected into the same EP tube. For experimental accuracy, three replicated wells were included for each group. This method ensured the isolation of high‐purity dNK cells and HTR‐8/SVneo cells for subsequent analyses.

### 2.5 Reverse Transcription Quantitative Polymerase Chain Reaction (RT‐qPCR)

As previously reported [18], total RNA was extracted using the Trizol method (Invitrogen) and reverse transcribed into complementary DNA using the PrimeScript RT‐PCR kit (Takara, Tokyo, Japan) following the manufacturer's instructions. miRNA quantification was performed on an ABI Prism 7500 Detection System (Applied Biosystems, Foster City, CA, USA) using the SYBR Premix Ex TaqTM kit (Takara). The qPCR reaction was conducted in a 20 μL volume under the following cycling conditions: pre‐denaturation at 95°C for 5 min, followed by 40 cycles of denaturation at 95°C for 10 s and annealing at 60°C for 30 s. U6 was used as the internal reference for miR‐152 expression. Relative gene expression was calculated using the 2^−ΔΔct^ method. The primer sequences are provided in Table 1.

**TABLE 1:** Primer sequences.

Gene	Forward primer (5′‐3′)	Reverse primer (5′‐3′)
miR‐152	GTCGTCAGTGCATGACAGAACTT	GTGCAGGGTCCGAGGT
U6	GTGCTCGCTTCGGCAGCACATATA	AATATGGAACGCTTCACGAATT

### 2.6 Cell Counting Kit‐8 (CCK‐8) Assay

Following the manufacturer's instructions, the CCK‐8 (Dojindo Molecular Technologies, Gaithersburg, MD, USA) was utilized to assess HTR‐8/SVneo cell viability [19]. Based on the previously described cell treatments and grouping, cells were seeded into a 96‐well plate (2 × 10^3^ cells per well) and cultured in RPMI‐1640 medium supplemented with 10% FBS (Thermo Fisher Scientific). At 24, 48, and 72 h, suspended dNK cells were isolated and 10 μL of CCK‐8 reagent was added to each well. After incubation at 37°C for 3 h, absorbance at 450 nm was measured using a Microplate Reader (Bio‐Rad, Hercules, CA, USA) to quantify cell viability.

### 2.7 Cell Apoptotic Assay

As previously mentioned [19], HTR‐8/SVneo cell apoptosis was evaluated using the Annexin V‐FITC Apoptosis Detection kit (BD Biosciences) as previously described. Suspended dNK cells were removed, and HTR‐8/SVneo cells were collected, resuspended in 500 μL 1 × Binding buffer, and stained with 5 μL Annexin V‐FITC and 5 μL PI (50 μg/mL). After incubation at room temperature in the dark for 15 min, apoptosis was quantified via FCM (BD Biosciences).

### 2.8 Transwell Assay

HTR‐8/SVneo cell invasion was evaluated using a Transwell assay, as described previously [13]. HTR‐8/SVneo cells (containing dNK cell co‐culture supernatant) were collected following different treatments. A total of 200 μL of cell suspension (5 × 10^5^ cells/mL) was seeded into the upper chamber of a Transwell insert, positioned in a 24‐well plate containing 500 μL RPMI 1640 with 10% FBS. The plates were cultured with 5% CO_2_ in the air at 37°C for 24 h. Non‐invading cells were removed using cotton swabs, while the invaded cells were fixed with 4% paraformaldehyde (Beyotime, Shanghai, China) and stained with 0.1% crystal violet (Beyotime). Invasion was quantified by analyzing non‐overlapping fields at 100 × magnification under a light microscope.

### 2.9 Dual‐Luciferase Report Assay

The potential binding sites of miR‐152 to HLA‐G were predicted using the RNAhybrid 2.2 database (https://bibiserv.cebitec.uni‐bielefeld.de/rnahybrid). Based on the predicted binding sequences, both the complementary binding sequence and mutation sequence of miR‐152 with HLA‐G were amplified and cloned into the pGL3 luciferase vector (Promega, Madison, WI, USA). This generated wild‐type plasmid HLA‐G‐WT and the corresponding mutant plasmid HLA‐G‐MUT. As previously described [20], mimics NC or miR‐152 mimics, inhibitor NC or miR‐152 inhibitor were transfected with HLA‐G‐WT plasmid or HLA‐G‐MUT plasmid (GenePharma) into HTR‐8/SVneo cells using LipofectamineTM 2000 (Invitrogen) following the manufacturer's instructions. After 48 h, luciferase activity was measured using the Dual‐Luciferase Reporter Assay System (Promega). This dual‐luciferase reporter assay was used to confirm the targeted binding between miR‐152 and HLA‐G.

### 2.10 Western Blot

The total protein was extracted using radio‐immunoprecipitation assay lysis buffer (Beyotime) containing a protease inhibitor (Roche, Complete Mini, Basel, Switzerland), following the manufacturer's instructions. Protein concentration was determined using the bicinchoninic acid test kit (Beyotime). Subsequently, 50 μg protein was loaded onto a 10% sodium dodecyl sulfate (SDS) polyacrylamide gel electrophoresis and transferred onto polyvinylidene fluoride membranes (Millipore, Billerica, MA, USA). The membranes were blocked with Tris‐buffered saline with Tween 20 (TBST) (Beyotime) containing 5% skim milk at room temperature, then incubated overnight at 4°C with primary antibodies Bax (1:1000, ab32503, Abcam, Cambridge, UK), Bcl‐2 (1:2000, ab182858, Abcam), HLA‐G (1:500, MEM‐G/1, ab7759, 40kDA, Abcam) and tubulin (GTX11325, GeneTex, CA, USA). After washing, the membranes were incubated with secondary antibody goat anti‐mouse IgG H&L (HRP) (1:2000, ab205719, Abcam) for 1 h. Protein bands were detected using enhanced chemiluminescence (Seyotin, Guangzhou, Guangdong, China), and the gray values were analyzed using ImageJ software (National Institutes of Health, Bethesda, MD, USA).

### 2.11 Enzyme‐Linked Immunosorbent Assay (ELISA)

The cell supernatant from each group was collected for cytokine analysis, which included determining the secretion of key immune‐regulatory molecules by dNK cells [21]. To assess cytokine levels, the Luminex Human Discovery Assay (3‐Plex) kit (LXSAHM‐03, R&D systems, Minneapolis, MN, USA) was employed, which includes beads conjugated with monoclonal antibodies targeting VEGF, IFN‐IP‐10, and IL‐8 [13]. The procedure was carried out according to the manufacturer's instructions to ensure accurate quantification. Additionally, the secreted sHLA‐G levels in the supernatant were measured using the Human MHCG/HLA‐G ELISA Kit (E‐EL‐H1663, Elabscience, Wuhan, Hubei, China). The kit has a detection range of 0.63–40 ng/mL, a sensitivity threshold of 0.38 ng/mL, and demonstrated high specificity with negligible cross‐reactivity with similar analogs. For both assays, the intra‐ and inter‐plate coefficients of variation were determined to be less than 10%, indicating robust repeatability. All procedures were meticulously followed to the manufacturer's instructions. Following incubation with the substrate for 30 min, absorbance was recorded at 450 nm using a Benchmark microplate reader (Bio‐Rad) to quantify cytokine and sHLA‐G levels.

### 2.12 Co‐Immunoprecipitation

To examine the binding of HLA‐G and KIR2DL4, co‐immunoprecipitation was conducted using dNk cell lysates [22]. The cells were first harvested and lysed in IP buffers (Beyotime) containing phenylmethylsulfonyl fluoride protease inhibitor to prevent protease activity. A total of 30 μL of the whole‐cell extract was set aside as input for the assay, while the remaining lysate was divided into two parts for antibody incubation. Each part of the lysate was incubated with either an anti‐KIR2DL4 (GTX80038, GeneTex) or an anti‐HLA‐G (ab4570, Abcam) antibody overnight at 4°C. An IgG control (GTX35009, GeneTex) was also used in parallel to account for any non‐specific binding. During incubation, the samples were gently rotated to facilitate antibody binding. Subsequently, protein G magnetic beads (Invitrogen) were added to the lysates and incubated for 2–3 h, as per the manufacturer's instructions, to capture the antibody‐bound protein complexes. The beads were washed five times with TBST to remove unbound proteins. The bound proteins were eluted by boiling the beads in 1 × SDS sample buffer for 5 min. Finally, both the input whole‐cell extracts and the eluted proteins were analyzed by Western blot to detect and confirm the binding interaction between HLA‐G and KIR2DL4.

### 2.13 Statistical Analysis

Data were analyzed and presented using GraphPad Prism 8.01 (GraphPad Software, San Diego, CA, USA) software. Measurement data were reported as mean ± standard deviation (SD). An independent sample *t*‐test was employed for comparisons between two groups. For multi‐group comparisons, one‐way analysis of variance (ANOVA) was performed, followed by Tukey's multiple comparisons test to assess pairwise differences. All statistical tests were two‐tailed, and a *p*‐value less than 0.05 (*p <* 0.05) was considered significant.

## 3 Results

### 3.1 miR‐152 Suppressed dNK Cell Secretory Function and HTR‐8/SVneo Cell Invasive Ability

**FIGURE 1:**
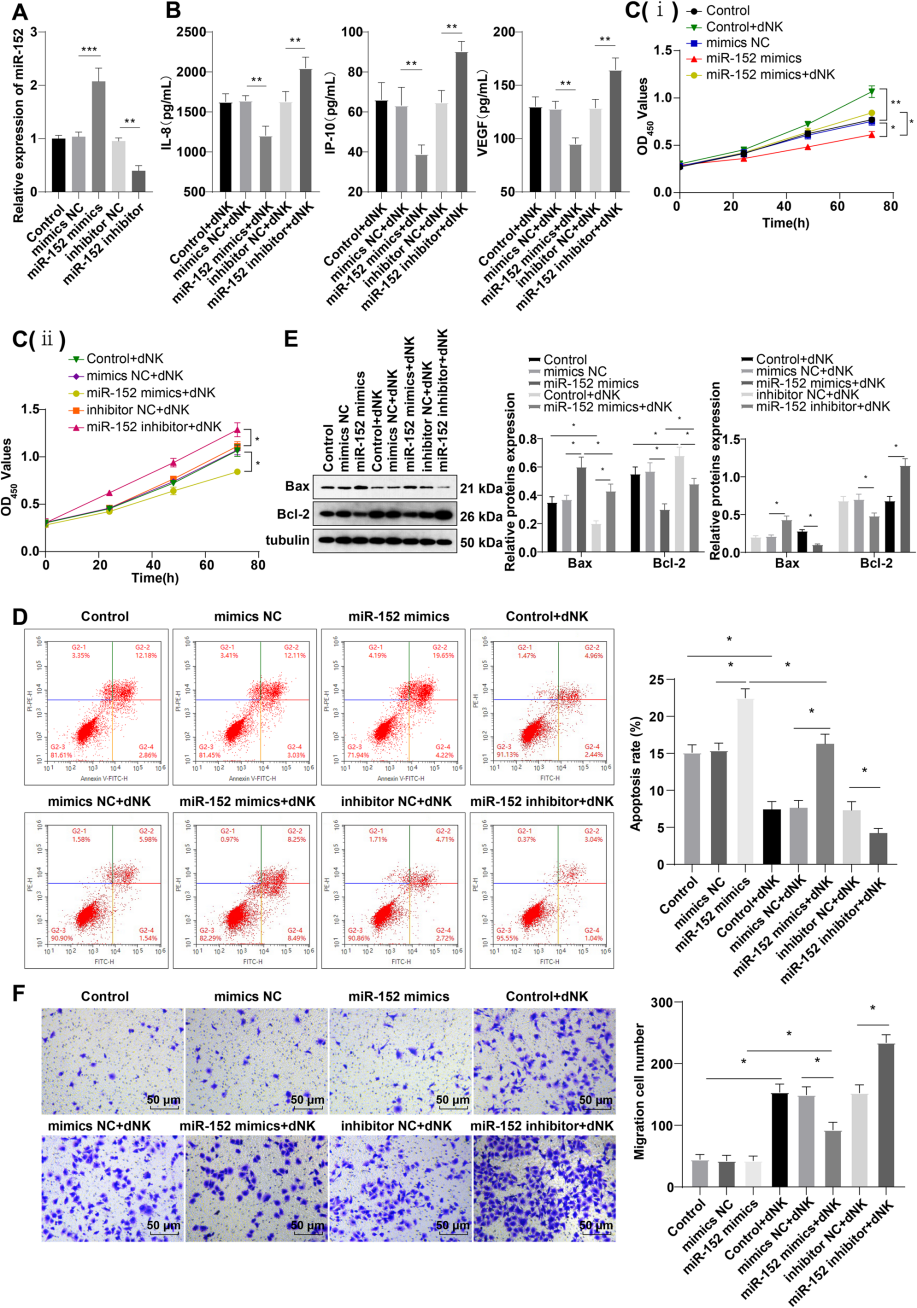
miR‐152 inhibited dNK cell secretory function and invasive ability of HTR‐8/Svneo cells. (A) RT‐qPCR detected miR‐152 transfection efficiency; (B) Luminex Human Discovery Assay (3‐Plex) kit was utilized to determine the levels of dNK secretory cytokines (IL‐8, IP‐10, VEGF) in the supernatant; C(i) and C(ii): CCK‐8 was used to analyze HTR‐8/Svneo cell viability; (D) FCM was used to evaluate HTR‐8/Svneo cell apoptosis; (E) Western blot to examine the levels of pro‐apoptotic protein Bax and anti‐apoptotic protein Bcl‐2; (F) Transwell determined HTR‐8/Svneo cell invasion. The cell experiment was repeated three times. Data were expressed as mean ± standard deviation. One‐way ANOVA was adopted for comparisons, followed by Tukey's test. *p < 0.05, **p < 0.01, ***p < 0.001.

To assess the effects of miR‐152 on dNK cell secretory activity and HTR‐8/SVneo cell invasion, HTR‐8/SVneo cells were transfected with miR‐152‐mimics/inhibitor and their respective negative controls (mimics NC and inhibitor NC). RT‐qPCR results confirmed the successful introduction of miR‐152‐mimics/inhibitor into the cells (Figure 1A, all *p* < 0.01). A co‐culture system of dNK cells and HTR‐8/SVneo cells was then established. Cytokine analysis showed no significant difference in the levels of IL‐8, IP‐10, and VEGF between the Control + dNK, mimics NC + dNK, and inhibitor NC + dNK groups (Figure 1B, *p* > 0.05). In contrast, the miR‐152 mimics + dNK group exhibited significantly lower levels of IL‐8, IP‐10, and VEGF compared to the mimics NC + dNK group (Figure 1B, *p* < 0.01). Additionally, in the miR‐152 inhibitor + dNK group, the levels of IL‐8, IP‐10, and VEGF were significantly increased compared to the inhibitor NC + dNK group (Figure 1B, *p* < 0.01), providing evidence that miR‐152 suppresses the secretory function of dNK cells.

The miR‐152 mimics group showed reduced HTR‐8/Svneo cell viability and anti‐apoptotic protein Bcl‐2 level, along with an increased cell apoptotic rate and pro‐apoptotic protein Bax level when compared to the mimics NC group (Figure 1C–E, all *p* < 0.05). However, there was no significant change in cell invasive ability (Figure 1F, *p* > 0.05). Compared to the Control group, the Control + dNK group showed substantial increases in HTR‐8/Svneo cell viability, invasive ability, and Bcl‐2 level, accompanied by significant decreases in apoptotic rate and Bax protein levels (Figure 1C–F, all *p* < 0.05). These findings suggest that miR‐152 decreases HTR‐8/Svneo cell viability, induces apoptosis, and has no significant impact on invasion. In contrast, dNK cells enhance HTR‐8/Svneo cell viability, reduce apoptosis, and promote cell invasion.

There were no significant differences in cell viability, apoptosis, Bcl‐2 and Bax protein levels, or invasion ability among the Control + dNK group, mimics NC + dNK group, and inhibitor NC + dNK group (Figure 1C–F, *p* > 0.05). However, compared to the mimics NC + dNK group, the miR‐152 mimics + dNK group showed marked reductions in HTR‐8/SVneo cell viability, invasive capacity, and Bcl‐2 expression, while exhibiting elevated apoptotic rate and Bax level (Figure 1C–F, all *p* < 0.05). In comparison to the miR‐152 mimics group, the miR‐152 mimics + dNK group showed increased viability and invasion and a decreased apoptotic rate. Meanwhile, the miR‐152 inhibitor + dNK group displayed significantly enhanced cell viability, Bcl‐2 expression, and invasion capacity and notably lower apoptotic rate and Bax levels compared to the inhibitor NC + dNK group (Figure 1C–F, *p* < 0.05). These results suggest that miR‐152 may regulate the proliferation, apoptosis, and invasion of HTR‐8/Svneo cells through modulating the secretory function of dNK cells. Overall, the data highlight that miR‐152 suppresses dNK cell secretory function and HTR‐8/SVneo cell invasion.

### 3.2 miR‐152 Targeted to Restrain HLA‐G Expression in HTR‐8/SVneo Cells

**FIGURE 2:**
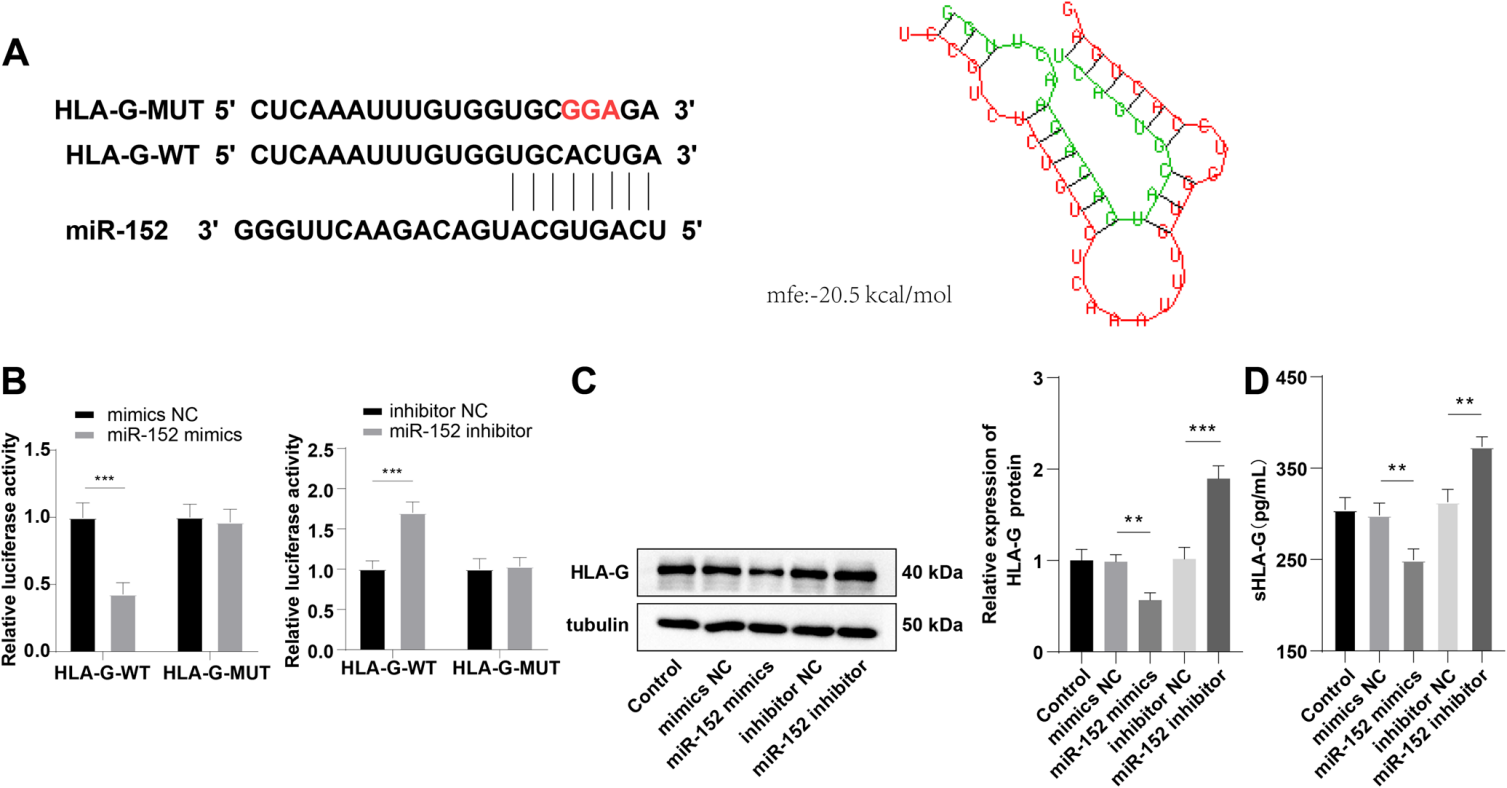
miR‐152 targeted HLA‐G to repress its expression in HTR‐8/Svneo cells. (A) Referring to the reported potential binding sites between miR‐152 and HLA‐G, (B) We conducted a dual‐luciferase reporter assay to verify their binding relationship; (C) Western blot was used to assess the level of HLA‐G protein; (D) ELISA measured sHLA‐G level in the supernatant. The cell experiment was repeated thrice. Data were expressed as mean ± standard deviation. In panel B, an independent sample *t*‐test was applied for comparisons between the two groups, one‐way ANOVA was employed for comparisons among multiple groups in panels C–D, followed by Tukey's multiple comparisons test. **p < 0.01, ***p < 0.001.

miR‐152 has been identified as a regulator of HLA‐G expression [18]. Using the RNAhybrid 2.2 database, we predicted potential binding sites between miR‐152 and HLA‐G (Figure 2A). To validate this prediction, we conducted a dual‐luciferase reporter assay to determine whether miR‐152 directly interacts with HLA‐G. The results showed that the relative luciferase activity significantly decreased upon co‐transfection of miR‐152 mimics and HLA‐G‐WT plasmid, whereas co‐transfection with miR‐152 inhibitors led to a significant increase in luciferase activity (Figure 2B, *p* < 0.001). In contrast, co‐transfection of miR‐152 mimics/inhibitors with HLA‐G‐MUT did not affect luciferase activity (Figure 2B, *p* > 0.05) confirming the specificity of miR‐152 binding to HLA‐G. To further assess the effect of miR‐152 on HLA‐G expression, we analyzed HLA‐G protein levels in cells and sHLA‐G in the culture supernatant by Western blot and ELISA. No significant variations were observed among the Control, mimics NC, and inhibitor NC groups (Figure 2C,D, *p* > 0.05). However, cells transfected with miR‐152 mimics exhibited significantly lower levels of HLA‐G and sHLA‐G (Figure 2C,D, all *p* < 0.01), whereas miR‐152 inhibition resulted in a marked increase in these levels (Figure 2C,D, all *p* < 0.01). The findings provide strong evidence that miR‐152 negatively regulates HLA‐G expression in HTR‐8/SVneo cells by directly binding to its target site.

### 3.3 Upregulation of HLA‐G Partially Reversed the Inhibitory Effect of miR‐152 on dNK Cell Secretory Function

**FIGURE 3:**
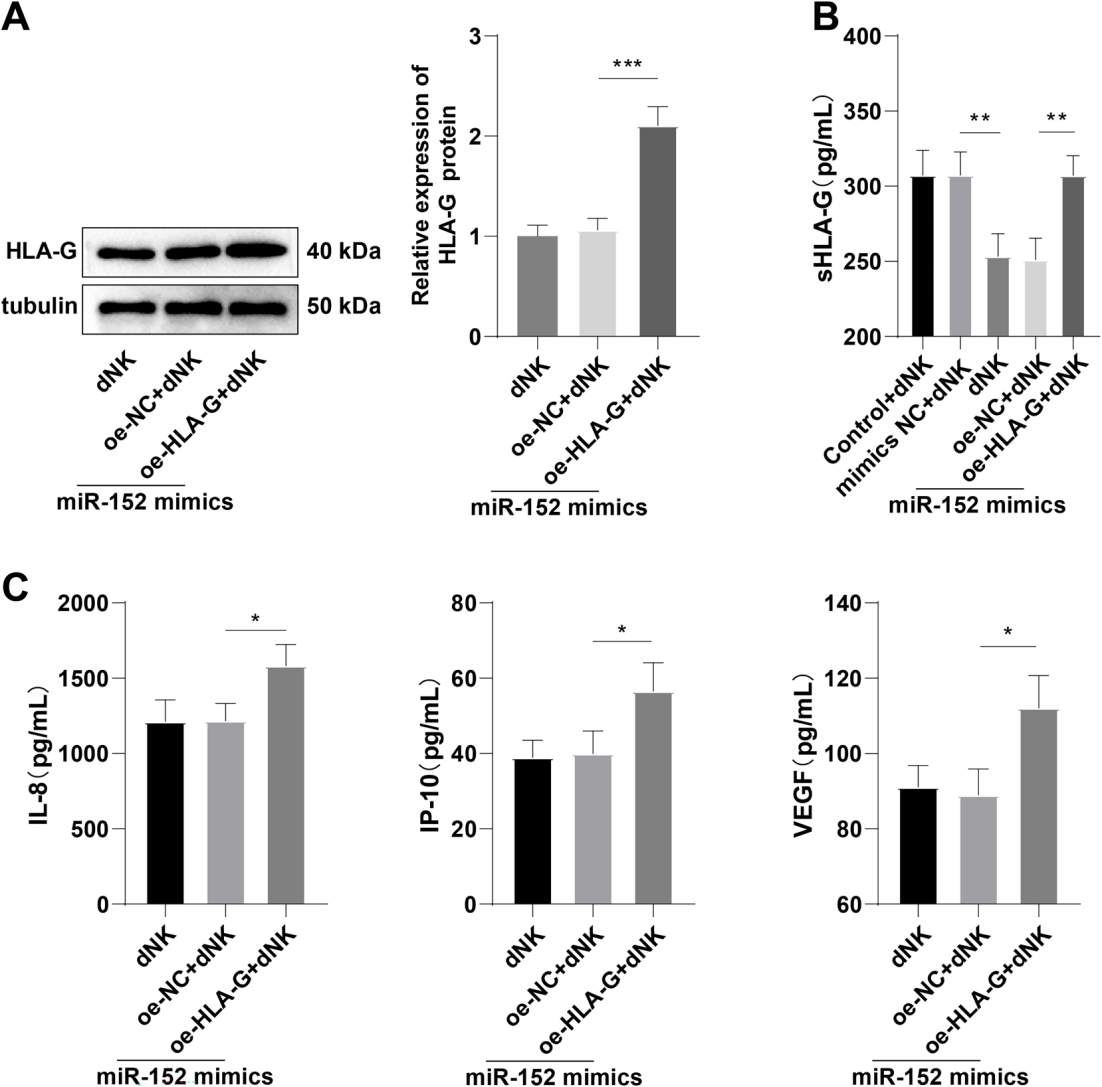
Upregulation of HLA‐G partially averted the inhibitory effect of miR‐152 on dNK cell secretory function. (A) After cell transfection, western blot was used to assess the level of HLA‐G protein; (B) ELISA determination of sHLA‐G level in cell supernatant; (C) The Luminex Human Discovery Assay (3‐Plex) kit assessed the levels of dNK secretory cytokines (IL‐8, IP‐10, VEGF) in the supernatant. The cell experiment was repeated three times. Data were expressed as mean ± standard deviation. One‐way ANOVA was applied for comparisons, followed by Tukey's multiple comparisons test. *p < 0.05, **p < 0.01, ***p < 0.001.

To investigate whether the miR‐152/HLA‐G axis influences the secretory function of dNK cells, we first co‐transfected HTR‐8/SVneo cells with miR‐152 mimics and oe‐HLA‐G and subsequently co‐cultured these cells with dNK cells. Western blot analysis was performed to verify the expression of HLA‐G in different experimental conditions. The results showed that the HLA‐G protein level did not differ significantly between the miR‐152 mimics + dNK group and the miR‐152 mimics + oe‐NC + dNK group (Figure 3A, all *p* > 0.05). However, a substantial increase was observed in the miR‐152 mimics + oe‐HLA‐G + dNK group compared to the miR‐152 mimics + oe‐NC + dNK group (Figure 3A *p* < 0.001), indicating that oe‐HLA‐G transfection was successful and effectively restored HLA‐G expression.

Moreover, ELISA analysis demonstrated that sHLA‐G levels in the cell supernatant were comparable between the Control + dNK and mimics NC + dNK groups, as well as between miR‐152 mimics + dNK and miR‐152 mimics+oe‐NC + dNK groups (Figure 3B, all *p* > 0.05). However, the sHLA‐G levels in the mimics NC + dNK group were significantly lower than those in the miR‐152 mimics + dNK group (Figure 3B, all *p* < 0.01). Conversely, the miR‐152 mimics + oe‐NC + dNK group exhibited a notable increase in sHLA‐G levels compared to the miR‐152 mimics + oe‐HLA‐G + dNK group (Figure 3B, all *p* < 0.05). Further quantification of IL‐8, IP‐10, and VEGF levels in the supernatant revealed no notable differences between the miR‐152 mimics + dNK and miR‐152 mimics+oe‐NC + dNK groups (Figure 3C, *p* > 0.05). However, IL‐8, IP‐10, and VEGF levels were significantly higher in the miR‐152 mimics + oe‐HLA‐G + dNK group than in miR‐152 mimics+oe‐NC + dNK group (Figure 3C, all *p* < 0.05). These results indicate that increased HLA‐G expression can at least partially reverse the inhibitory effect of miR‐152 on dNK cell secretory activity.

### 3.4 Increased Expression of HLA‐G Partly Counteracted miR‐152‐Mediated Inhibitory Influence on the Invasive Ability of HTR‐8/SVneo Cells

**FIGURE 4:**
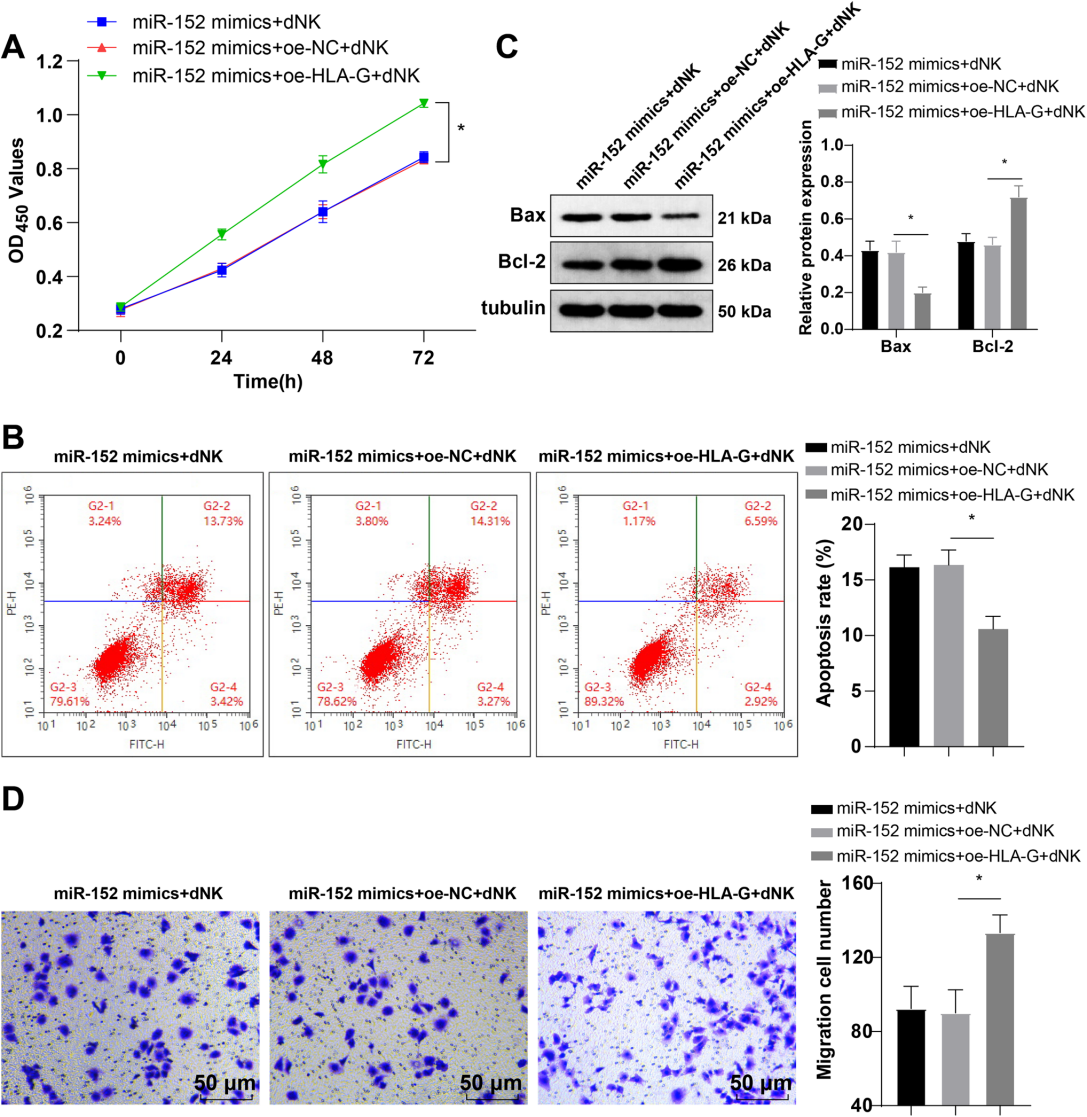
HLA‐G upregulation partially annulled the inhibitory effect of miR‐152 on the invasion of HTR‐8/Svneo cells. (A) CCK‐8 evaluated HTR‐8/Svneo cell viability; (B) FCM was used to assess HTR‐8/Svneo cell apoptosis; (C) Measurement of protein Bax and Bcl‐2 levels by Western blot; (D) Transwell evaluated HTR‐8/Svneo cell invasion. The cell experiment was repeated thrice. Data were expressed as mean ± standard deviation. One‐way ANOVA was used for comparisons, followed by Tukey's multiple comparisons test. *p < 0.05.

To determine the role of the miR‐152/HLA‐G axis in regulating HTR‐8/SVneo cell invasion, we co‐transfected HTR‐8/SVneo cells with miR‐152 mimics and oe‐HLA‐G, and then co‐cultured them with dNK cells. Evaluation of cell viability, apoptosis, and invasion using CCK‐8, FCM, Western blot, and Transwell assays demonstrated no marked differences between miR‐152 mimics + dNK and miR‐152 mimics + oe‐NC + dNK groups (Figure 4A–D, *p* > 0.05). However, the miR‐152 mimics+oe‐HLA‐G + dNK group exhibited a substantial increase in Bcl‐2 level, cell viability, and invasive ability, along with a significant reduction in Bax expression and apoptosis compared to the miR‐152 mimics+oe‐NC + dNK group (Figure 4A–D, all *p* < 0.05). These results indicate that elevated HLA‐G expression partially mitigates the inhibitory effect of miR‐152 on HTR‐8/SVneo cell invasion.

### 3.5 Blocking HLA‐G/KIR2DL4 Interaction Partially Averted miR‐152 Knockout‐Induced Promotive Effects on dNK Cell Secretory Function and HTR‐8/SVneo Cell Invasion

**FIGURE 5:**
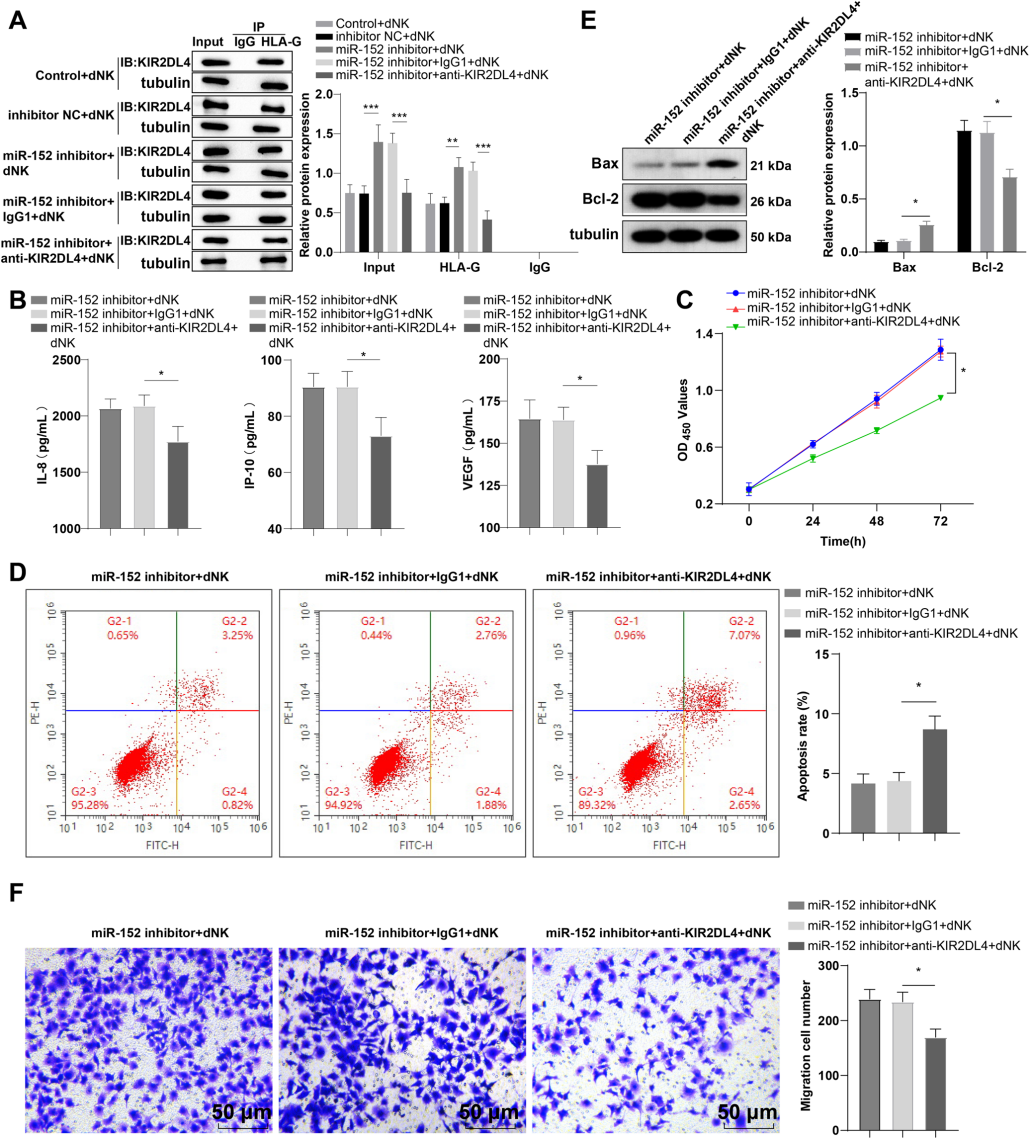
Blocking HLA‐G/KIR2DL4 binding partially abrogated miR‐152 knockout‐mediated promotion on dNK cell secretory function and HTR‐8/Svneo cell invasion. (A) The interaction between HLA‐G and KIR2DL4 was detected by co‐immunoprecipitation; (B) Luminex human discovery assay (3‐Plex) kit was used to determine the levels of dNK secretory cytokines (IL‐8, IP‐10, VEGF) in the supernatant; (C) CCK‐8 detection of HTR‐8/Svneo cell viability; (D) FCM was used to assess HTR‐8/Svneo cell apoptosis; (E) Detection of Bcl‐2 and Bax levels by Western blot; (F) Transwell evaluates cell invasion. The cell experiment was repeated thrice. Data were expressed as mean ± standard deviation. One‐way ANOVA was used for comparisons, followed by Tukey's multiple comparisons test. *p < 0.05, **p < 0.01, ***p < 0.001.

To gain deeper insight into how miR‐152 modulates dNK cell secretory function and HTR‐8/SVneo cell invasion via the HLA‐G/KIR2DL4 axis, we treated the co‐culture medium of dNK cells and HTR‐8/SVneo cells (transfected with miR‐152‐inhibitor) with 10 μg/mL anti‐KIR2DL4/IgG1 to prevent HLA‐G/KIR2DL4 interaction. Co‐immunoprecipitation assays verified the successful blockade of HLA‐G/KIR2DL4 binding (Figure 5A). No significant changes were observed in the secretion of IL‐8, IP‐10, and VEGF or in HTR‐8/Svneo cell apoptosis, invasion, and viability between the miR‐152 inhibitor + dNK and miR‐152 inhibitor+IgG1 + dNK groups (Figure 5B–F, *p* > 0.05). However, compared to the miR‐152 inhibitor + IgG1 + dNK group, the miR‐152 inhibitor + anti‐KIR2DL4 + dNK group exhibited distinctly reduced levels of IL‐8, IP‐10, and VEGF (Figure 5B, all *p* < 0.05), along with a marked decline in HTR‐8/SVneo cell viability and invasion, an increase in apoptotic rate, enhanced Bax level, and suppressed Bcl‐2 level (Figure 5C–F, all *p* < 0.05). These findings suggest that the pro‐secretory and pro‐invasive effects resulting from miR‐152 downregulation were partially counteracted by preventing the binding of HLA‐G to KIR2DL4.

## 4 Discussion

PE is a leading cause of preterm birth, and its untreated progression poses significant risks to maternal and fetal health [23]. Recent evidence has demonstrated that various miRNAs contribute to the pathophysiology of PE by modulating trophoblast invasion [24, 25]. In this study, we identified miR‐152 as a regulatory factor that directly targets HLA‐G, thereby reducing its interaction with KIR2DL4. This interference attenuated the secretory function of dNK cells and restricted the invasive capacity of HTR‐8/SVneo trophoblast cells. The novel contributions of this study are as follows: (1) It provides the evidence that miR‐152 in HTR‐8/Svneo trophoblast cells inhibits cytokine secretion by dNK cells via HLA‐G; (2) It establishes a mechanistic link between miR‐152 and trophoblast invasion, mediated by dNK cell regulation within the endometrial microenvironment; (3) It delineates the role of the HLA‐G/KIR2DL4 axis in modulating dNK cell cytokine secretion, thereby elucidating a previously uncharacterised mechanism through which miR‐152 inhibits trophoblast invasion.

dNK cells possess limited cytolytic capacity and contribute to placental development, trophoblast invasion, embryonic development, and vascular remodeling primarily through cytokine and chemokine secretion [7]. Given their immunomodulatory function, investigating the effect of trophoblasts on dNK cytokine production may provide crucial insights into maternal‐fetal immune interactions. Existing studies have demonstrated that miR‐122‐5p downregulation impairs HTR‐8/SVneo cell invasion, thereby enhancing inflammatory cytokine secretion by dNK cells [26]. Additionally, up‐regulated miR‐30e attenuates the cytotoxicity of both peripheral blood natural killer (PB‐NK) cells and dNK cells via PRF1 targeting, subsequently shifting the immune balance from a Th1 tolerance phenotype to Th2 immunodominance. This suggests a pivotal role for miR‐30e in fostering immune tolerance at the maternal‐fetal interface [27]. Aberrant up‐regulation of miR‐152 has been implicated in PE, highlighting its potential as a predictive serum biomarker [28]. However, its specific role in dNK cell secretory function and trophoblast cell invasion remains underexplored. Notably, Xiao‐ming Zhu et al. reported that miR‐152 overexpression suppresses HLA‐G expression, thereby increasing NK cell‐mediated cytolysis in JEG‐3 trophoblast cells; although it does not directly affect their invasive potential [18]. In contrast, our study demonstrates that miR‐152 inhibits dNK cell secretory function, leading to the suppression of trophoblast HTR‐8/SVneo cell invasion, proliferation, and apoptosis. Furthermore, previous research indicates that progesterone‐induced miR‐152 downregulates GLUT3 in endometrial epithelial cells, disrupting early embryonic development and implantation [29]. Additionally, miR‐152 has been shown to improve hepatic insulin resistance in gestational diabetes mellitus (GDM) mouse models by downregulating SOCS3 expression [30]. These findings collectively suggest that miR‐152 may represent a viable therapeutic target for PE.

Under physiological conditions, HLA‐G is highly and specifically expressed on extravillous trophoblasts, suggesting a potential role in pregnancy‐related complications [31]. KIR2DL4, a specific receptor for HLA‐G, is ubiquitously expressed in all NK cells and plays a critical role in modulating immune responses [11]. The HLA‐G/KIR2DL4 interaction has been shown to facilitate dNK cell cytokine secretion while simultaneously suppressing their cytotoxicity, thereby promoting trophoblast cell invasion and maintaining local immune suppression [32]. Therapeutic strategies targeting inhibitory NK cell receptors and HLA molecules have been explored as potential approaches for modulating immune responses [22]. Previous studies have demonstrated that blocking the HLA‐G interaction with LILRB1 suppresses IFN‐γ and VEGFα secretion in dNK cells [33]. Fu et al. reported that inhibition of the HLA‐G‐ILT2‐KIR2DL4 pathway disrupts NK cell‐mediated secretion of growth‐promoting factors [34]. Consistent with these findings, our study demonstrated that blocking the HLA‐G/KIR2DL4 axis following miR‐152 silencing resulted in suppressed dNK cell secretory function and reduced HTR‐8/SVneo cell invasion. HLA‐G has been previously identified as a direct target of miR‐152 [35], a relationship validated in our study through dual luciferase reporter assay. This interaction has also been observed in human bronchial epithelial cell lines and extravillous cytotrophoblasts [36]. Our results further confirm that miR‐152 directly binds to and downregulates HLA‐G expression in HTR‐8/SVneo cells [16]. Moreover, blockade of the HLA‐G/KIR2DL4 axis partially reversed the enhanced trophoblast invasion and dNK cytokine secretion induced by miR‐152 silencing, providing further mechanistic insight into miR‐152‐mediated immune regulation. These findings suggest that miR‐152 plays a crucial role in regulating dNK cytokine secretion through the HLA‐G/KIR2DL4 axis, contributing to the pathogenesis of PE and identifying miR‐152 as a potential therapeutic target. Consistent with our findings, miR‐133a has also been reported to downregulate HLA‐G expression in HTR‐8/SVneo cells. Furthermore, HLA‐G binding to KIR2DL4 has been shown to suppress dNK cell cytokine secretion, thereby modulating trophoblast invasion and angiogenesis [13]. These findings collectively emphasize the importance of miRNA‐mediated regulatory networks in PE pathogenesis and provide insights into the molecular mechanisms governing trophoblast invasion inhibiting in PE.

This study has several limitations. First, the investigation of miR‐152's role in inhibiting HTR‐8/SVneo cell invasion was conducted solely through in vitro experiments. Conventional in vitro invasion assays do not fully replicate the complex cellular interactions and microenvironmental factors critical for normal homeostasis, which may affect the extrapolation of these findings to in vivo conditions. Second, while the study analyzed miR‐152 influence on IL‐8, VEGF, and IP‐10 secretion, additional cytokines involved in dNK cell‐mediated trophoblast regulation were not explored. Moreover, the underlying molecular mechanisms connecting the HLA‐G/KIR2DL4 axis to trophoblast invasion remain insufficiently characterized. Previous research suggests that downstream pathways, such as the mitogen‐activated protein kinase (MAPK) pathway, plays a critical role in this process [37, 38]. For example, Guo et al. demonstrated that HLA‐G5 activates the ERK signaling pathway through ERK1/2 phosphorylation, and inhibiting of ERK signaling reverses HLA‐G5‐induced trophoblast invasion [14]. These findings indicate the necessity for further mechanistic studies. Additionally, this study lacks clinical data, limiting its direct applicability to patient care. While miR‐152 has been proposed as a biomarker for conditions such as ST‐segment elevation myocardial infarction, urological malignant tumors, cervical cancer, and colon cancer [39, 40], there is insufficient clinical evidence validating its role in pregnancy‐related disorders, particularly PE. Future research should prioritize clinical sample collection from PE patients to quantify miR‐152 expression and assess its potential diagnostic and prognostic value in PE.

## 5 Conclusions

This study reveals that miR‐152 inhibits dNK cell secretory function and HTR‐8/SVneo cell invasion by targeting HLA‐G and reducing HLA‐G/KIR2DL4 interaction. These findings contribute to understanding the pathogenesis of PE and may guide further therapeutic strategies.

## Conflicts of Interest

The authors declare no conflicts of interest.

## Data Availability

The data that support the findings of this study are available from the corresponding author upon reasonable request.
